# Lovastatin causes FaDu hypopharyngeal carcinoma cell death via AMPK-p63-survivin signaling cascade

**DOI:** 10.1038/srep25082

**Published:** 2016-04-28

**Authors:** Chia-Sheng Yen, Jung-Chien Chen, Yi-Fang Chang, Ya-Fen Hsu, Pei-Ting Chiu, Ching Shiue, Yu-Fan Chuang, George Ou, Ming-Jen Hsu

**Affiliations:** 1 Department of General Surgery, Chi-Mei Medical Center, Tainan, Taiwan; 2 Division of General Surgery, Department of Surgery, Min-Sheng General Hospital, Taoyuan, Taiwan; 3 Division of Hematology and Oncology, Department of Internal Medicine, MacKay Memorial Hospital, Taipei, Taiwan; 4 Division of General Surgery, Department of Surgery, Landseed Hospital, Taoyuan, Taiwan; 5 Graduate Institute of Medical Sciences, College of Medicine, Taipei Medical University, Taipei, Taiwan; 6 Department of Medicine, University of British Columbia, Vancouver, British Columbia, Canada; 7 Department of Pharmacology, School of Medicine, College of Medicine, Taipei Medical University, Taipei, Taiwan

## Abstract

Statins are used widely to lower serum cholesterol and the incidence of cardiovascular diseases. Growing evidence shows that statins also exhibit beneficial effects against cancers. In this study, we investigated the molecular mechanisms involved in lovastatin-induced cell death in Fadu hypopharyngeal carcinoma cells. Lovastatin caused cell cycle arrest and apoptosis in FaDu cells. Lovastatin increased p21^cip/Waf1^ level while the survivin level was decreased in the presence of lovastatin. Survivin siRNA reduced cell viability and induced cell apoptosis in FaDu cells. Lovastatin induced phosphorylation of AMP-activated protein kinase (AMPK), p38 mitogen-activated protein kinase (MAPK) and transcription factor p63. Lovastatin also caused p63 acetylation and increased p63 binding to survivin promoter region in FaDu cells. AMPK-p38MAPK signaling blockade abrogated lovastatin-induced p63 phosphorylation. Lovastatin’s enhancing effect on p63 acetylation was reduced in HDAC3- or HDAC4- transfected cells. Moreover, transfection of cells with AMPK dominant negative mutant (AMPK-DN), HDAC3, HDAC4 or p63 siRNA significantly reduced lovastatin’s effects on p21^cip/Waf1^ and survivin. Furthermore, lovastatin inhibited subcutaneous FaDu xenografts growth *in vivo*. Taken together, lovastatin may activate AMPK-p38MAPK-p63-survivin cascade to cause FaDu cell death. This study establishes, at least in part, the signaling cascade by which lovastatin induces hypopharyngeal carcinoma cell death.

Despite advances in anti-cancer drug development in the last decades, the over-all survival rate and prognosis in head and neck squamous cell carcinoma (HNSCC) patients remain largely unchanged. It is therefore an ongoing urgent need for novel drugs in the treatment of HNSCC. The 3-hydroxy-3-methylglutaryl-coenzyme A (HMG-CoA) reductase inhibitors statins are widely used in treating hyperlipidemia and reducing the risk of cardiovascular and cerebrovascular events^1^,^2^. It also exert anti-inflammatory, anti-proliferative and pro-apoptotic activities beyond their well known properties as lipid-lowering drugs^3^,^4^. Statins arrested growth or induced cell death in different types of cancers such as colorectal, prostate, breast and lung cancers, as well as HNSCC^5^,^6^,^7^,^8^. It is also reported that statins suppress angiogenesis, tumor invasion and metastasis in xenograft animal models^9^,^10^. Although the experimental and clinical data remained controversial, statins still attract considerable attention for its therapeutic value in the treatment of cancer^11^,^12^,^13^,^14^. Recent study demonstrated that statins reduce cancer-related mortality in patients with HNSCC^15^. Ma *et al*.^16^, also showed that lovastatin induced squamous cell carcinoma (SCC) cell death through targeting metabolic stress pathways. However, the precise mechanisms involved in statins-induced HNSCC cell death remain incompletely understood. A human HNSCC-derived cell line, FaDu, was thus used to investigate the mechanisms by which lovastatin induces HNSCC cell death.

Statins’s anti-tumor effects may attribute to multiple mechanisms. These include suppression of protein geranylgeranylation^17^, activation of mitochondria apoptotic pathway via regulating Bcl-2 family members and suppression of cell cycle progression^6^,^18^. Many lines of evidence demonstrated that histone deacetylases (HDACs) are mechanistically correlated with the pathogenesis of cancer^5^,^19^. Statins have been recently shown to interfere HDAC activity to inhibit cell proliferation and suppress *in vivo* tumor growth^6^,^20^. Understanding the statin’s anti-tumor mechanisms will aid in their proper application as anti-cancer agents in the future.

Inhibitor-of-apoptosis protein (IAP) family contributes to the aberrantly increased cell survival in tumor cells^21^,^22^. Survivin, the smallest IAP family member, is over-expressed in different types of cancers such as lung, breast, colorectal cancers and HNSCC, but is largely undetectable in normal adult tissues^23^,^24^,^25^. In cnacer patients, survivin expression has been associated with reduced survival rate and therapeutic resistance^25^. Survivin thus represents an attractive therapeutic target for cancer treatment^22^,^24^,^26^. We recently demonstrated that survivin down-regulation leads to colorectal cancer cell death^6^,^27^. Intriguingly, besides its role as an IAP, survivin also plays an essential role in modulating mitosis and cell division^23^,^28^. Many transcription factors such as STAT3 and Sp1 contribute to the induction of survivin^29^. However, tumor suppressor p53 and its related protein p63 may counteract Sp1 binding to the promoter region and, thereby, suppress survivin expression^6^.

In addition to survivin, p53 also regulates the expression of target genes including p21^cip/Waf1^ and Bax, leading to apoptosis or cell cycle arrest^30^. p63 and p73, two p53 family members, also exhibit anti-proliferative and apoptotic activities via regulating p53-responsive target genes^31^. The loss of p53 function are usually found in various types of human cancers^32^,^33^,^34^. In contrast, p63 is rarely mutated or deletion in cancers^35^. Recent study showed that p63 activation leads to p53-deficient cell death or increases the efficacy of chemotherapy^36^. It appears that p63 might be a rational target for cancer treatment. However, the casual role of p63 in attenuating tumor progression and its underlying mechanisms remain incomplete understood^37^. The FaDu cell is a p53-deficient HNSCC cell line^38^. Defective p53-mediated apoptotic response has been reported in FaDu cells^39^. Whether p63 signaling contributes to lovastatin’s actions in inducing Fadu hypopharyngeal carcinoma cell death will also be investigated.

## Results

### Lovastatin arrested cell cycle and induced apoptosis in FaDu cells

**Figure 1:**
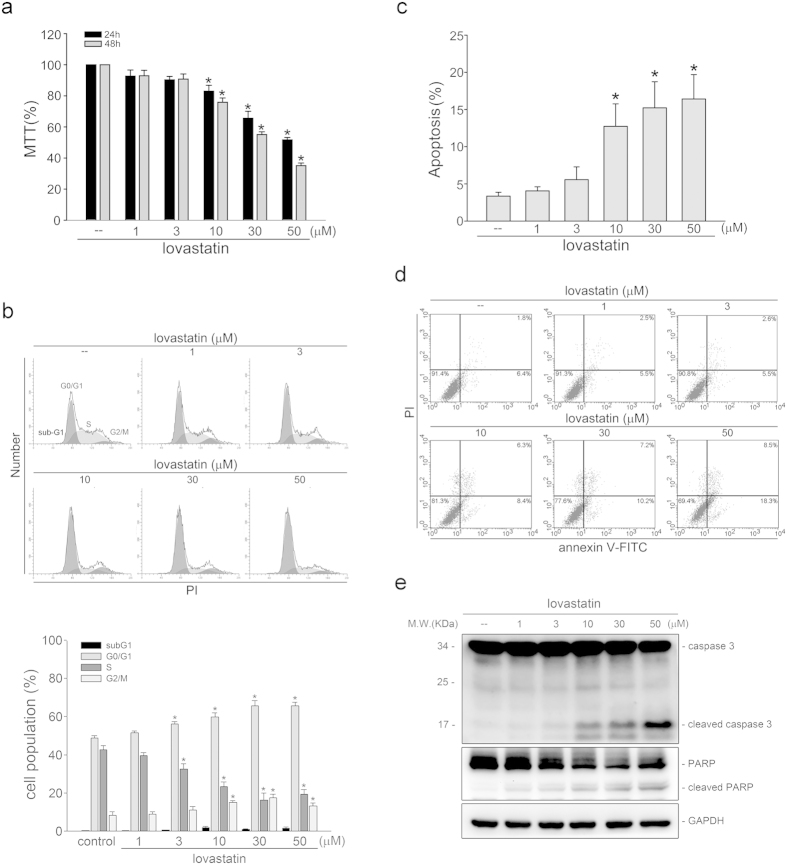
Lovastatin induced FaDu cell apoptosis. (**a**) After treatment with indicated concentrtions of lovastatin for 24 or 48 h, MTT assay was used to determine cell viability. Compiled results represent the mean ± S.E.M. of three independent experiments performed in duplicate. (**b**) After treatment with indicated concentrtions of lovastatin for 24 h, flow-cytometric analysis was used to analyze the cell cycle distribution. Compiled results are shown at the bottom (n = 7). (**c**) After treatment with indicated concentrtions of lovastatin for 48 h, Flow-cytometric analysis was used to determine the extent of cell apoptosis (subG1 region). Compiled results are shown at the bottom (n = 6). (**d**) Cells were treated as in (**c**). Flow-cytometric analysis with propidium iodide (PI) and annexin V-FITC double staining was used to determine the extent of cell apoptosis. Typical pattern shown are representative of three independent experiments. (**e**) After treatment as in (**c**), immunoblotting was then used to determine the cleavage caspase 3 and PARP levels. Typical pattern shown are shown are representative of four independent experiments. *p < 0.05, compared with the control group.

MTT assay was employed to determine whether FaDu cell viability is altered in the presence of lovastatin. As shown in Fig. 1a, lovastatin concentration-dependently decreased FaDu cell viability after 24 h exposure. Longer exposure to lovastatin (48 h) further decreased FaDu cell viability (Fig. 1a). To determine whether lovastatin-decreased FaDu cell viability was a result of cell cycle arrest or apoptosis, flowcytometry was used. As shown in Fig. 1b, the percentage of propidium iodide (PI)-stained cells in the S region was significantly decreased in FaDu cells after exposure to lovastatin for 24 h. In addition, lovastatin increased the percentage of PI-stained cells in the G0/G1 region (Fig. 1b). Moreover, 24 h treatment of lovastatin only slightly induced cell apoptosis (sub-G1 region) (Fig. 1b). However, lovastatin significantly induced apoptosis in FaDu cells after 48 h exposure of lovastatin (Fig. 1c). To detect apoptosis in FaDu cells exposed to lovastatin, flowcytometry with PI and annexin V-FITC double-labeling was also employed. As shown in Fig. 1d, lovastatin increased the percentage of early apoptotic cells (annexin V^+^PI^−^ cells) and advanced apoptotic cells and/or necrotic cells (annexin V^+^PI^+^ cells) after 48 h exposure. We next determined whether lovastatin activates caspase 3. As shown in Fig. 1e, lovastatin increased the cleaved (active) form of caspase 3 and PARP, a selective caspase 3 substrate. These findings suggest that lovastatin induced apoptosis and inhibited cell proliferation in FaDu cells.

### Lovastatin modulated p21^cip/Waf1^, cyclin D1 and survivin expressions in FaDu cells

**Figure 2:**
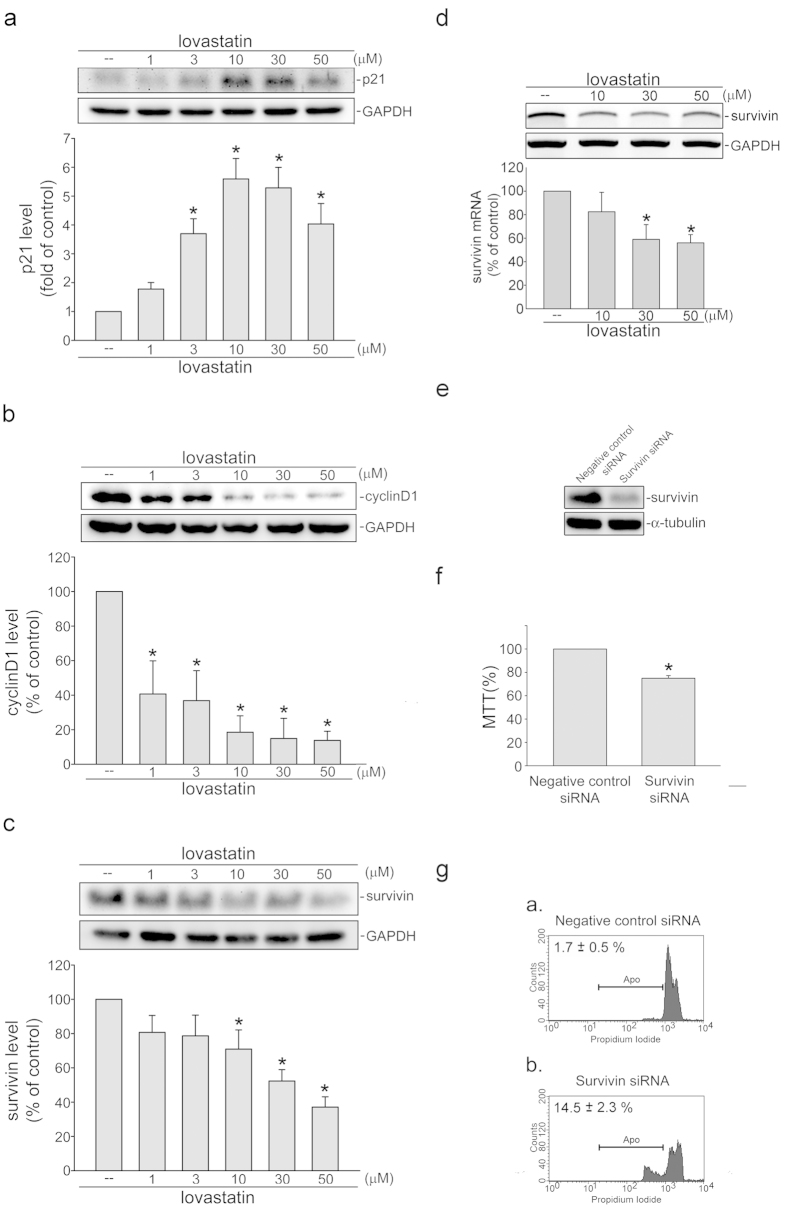
Lovastatin modulated p21^cip/Waf1^, survivin and cyclin D1 levels in FaDu cells. After 24 h treatment with indicated concentrations of lovastatin, the extent of p21^cip/Waf1^ (**a**), cycin D1 (**b**) and survivin (**c**) were assessed by immunoblotting. Compiled results represent the mean ± S.E.M. of at least six independent experiments (**d**) After 6 h treatment with indicated concentrations of lovastatin, RT-PCR analysis was used to determine the extent of *survivin mRNA*. Compiled results are shown at the bottom (n = 4). (**e**) After transfection, immunoblotting was used to determine the extent of survivin and α-tubulin. Results shown are representative of four independent experiments. (**f**) MTT assay was used to determine cell viability after transfection. Compiled results are shown at the bottom (n = 4). (**g**) Flow cytometric analysis was used to detect cell apoptosis after transfection. Typical pattern shown are representative of four independent experiments. *p < 0.05, compared with the control group.

Since cyclin-dependent kinase (CDK) inhibitor protein, p21^cip/Waf1^^40^, cyclin D1 and survivin^6^ play essential role in cell cycle progression or apoptosis. We therefore examined whether lovastatin had any effects on these proteins in FaDu cells. Results from immunoblotting analysis demonstrated that p21^cip/Waf1^ (Fig. 2a) was increased, while cycin D1 (Fig. 2b) and survivin (Fig. 2c) were decreased in FaDu cells exposed to lovastatin. We also determined whether lovastatin decreases survivin mRNA. Results from RT-PCR analysis demonstrated that lovastatin significantly decreased survivin mRNA in FaDu cells (Fig. 2d). A s*urvivin* siRNA oligonucleotide (s*urvivin* siRNA) was employed to determine whether survivin down-regulation induces FaDu cell apoptosis. Survivin siRNA reduced the basal surivvin level in FaDu cells (Fig. 2e). Survivin down-regulation by s*urvivin* siRNA mimicked the lovastatin’s effects in decreasing cell viability (Fig. 2f). Transfection with s*urvivin* siRNA also induced cell apoptosis (Fig. 2gb) while negative control siRNA was without effects (Fig. 2ga). These results suggest that reduced survivin level contributes to lovastatin-induced FaDu cell apoptosis.

### p63 contributes to lovastatin’s actions in FaDu cells

**Figure 3:**
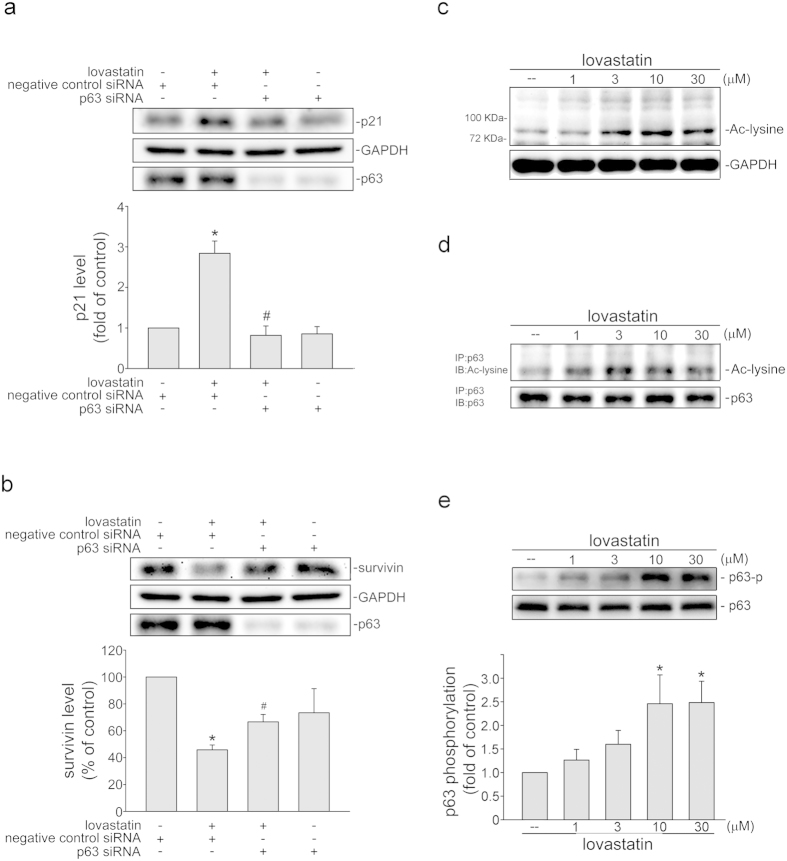
Lovastatin induced p63 phosphorylation and acetylation in FaDu cells. The extent of p21^cip/Waf1^ (**a**) and survivin (**b**) levels were assessed by immunoblotting after transfection. Compiled results are shown at the bottom (n = 4). (*p < 0.05, compared with the negative control siRNA group; ^#^p < 0.05, compared with the negative control siRNA group in the presence of lovastatin). (**c**) After 6 h treatment with lovastatin at indicated concentrations, the extent of acetylated protein was determined by immunoblotting with anti-acetyl-lysine antibody. Typical pattern shown are representative of four independent experiments. (**d**) After treatment as described in (**c**), total p63 was immunoprecipitated by anti-p63 antibody and the extent of p63 acetylation was determined by immunoblotting with anti-acetyl-lysine antibody. Typical pattern shown are representative of four independent experiments. (**e**) After treatment as described in (**c**), the extent of p63 phosphorylation was then assessed by immunoblotting. Compiled results are shown at the bottom (n = 5). *p < 0.05, compared with the control group.

Transcription factor p63 modulates several downstream target genes such as survivin and p21^cip/Waf1^^41^ ^6^, which regulate apoptosis and cell cycle progression. We therefore explored the impact of p63 on lovastatin’s actions in FaDu cells. As shown in Fig 3a, lovastatin-increased p21^cip/Waf1^ levels were reduced in FaDu cells transfected with p63 siRNA. p63 siRNA also reduced lovastatin’s effects on survivin levels (Fig. 3b). In addition, p63 siRNA markedly reduced the basal level of p63 in FaDu cells. Similar to p53, activation of p63 is modulated by its modifications such as acetylation and phosphorylation^42^,^43^,^44^. We thus determined the acetylated protein levels in FaDu cells after exposure to lovastatin. As shown in Fig. 3c, the acetylated protein level with molecular weights about 72 kDa (p63 molecular weight: 75 kDa) was increased in cells exposed to lovastatin as determined in immunoblotting using anti-acetylated lysine antibody (Cell Signaling). Resuls from immunoprecipitation analysis further confirmed that lovastatin induced p63 acetylation in FaDu cells (Fig. 3d). We also examined whether lovastatin induces p63 phosphorylation using anti-phosphorylated p63 antibody (Cell Signaling). Lovastatin significantly increased p63 phosphorylation at Ser160 and Ser162 in FaDu cells (Fig. 3e). Moreover, the anti-p40 antibody directed against an N-terminal truncated form of the p63 protein (ΔNp63) is currently replacing anti-p63 antibody as several studies^45^,^46^. To confirm p63 is truly involved in lovastatin’s actions in FaDu cells, anti-p40 antibody was used. Results from immunoblotting and siRNA experiments showed that anti-p63 antibody used in this study recognizes the same protein (p63) as anti-p40 antibody does (Supplementary Fig. 1). These results indicate that lovastatin treatment is capable of modulating p21^cip/Waf1^ and survivin and subsequent cellular events through, at least in part, activation of p63.

### HDACs inhibition contributes to lovastatin’s actions in FaDu cells

**Figure 4:**
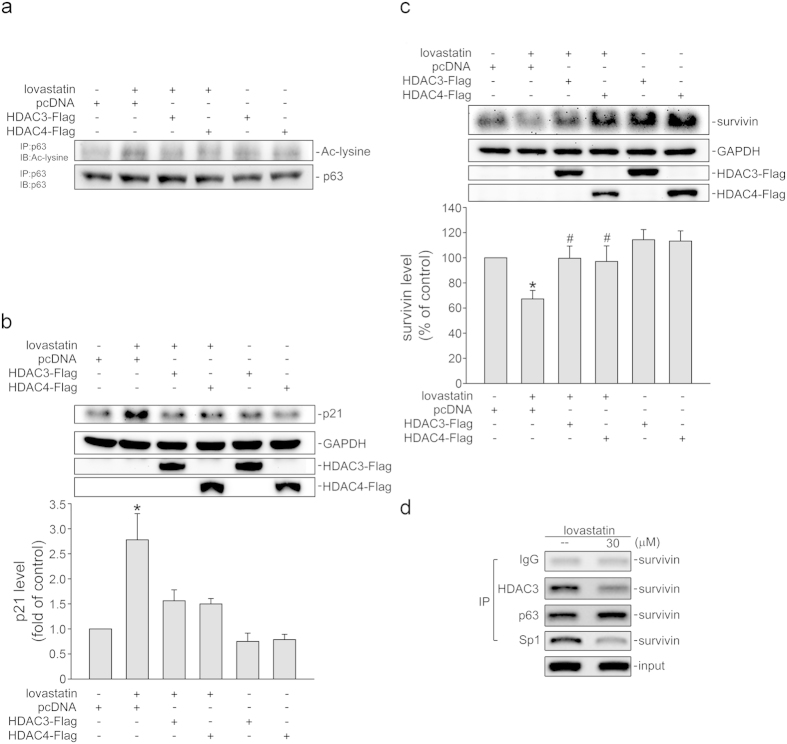
Lovastatin induced the recruitment of p63 to the survivin promoter region in FaDu cells. (**a**) After transfection, cells were treated with 30 μM lovastatin for another 6 h. Total p63 was immunoprecipitated by anti-p63 antibody and the extent of p63 acetylation was determined by immunoblotting with anti-acetyl-lysine antibody. Typical trace shown are representative of three independent experiments. After transfection, cells were treated with 30 μM lovastatin for another 24 h. The extent of p21^cip/Waf1^ (**b**) and survivin (**c**) were then assessed by immunoblotting. Compiled results are shown at the bottom (n = 6). (*p < 0.05, compared with the pcDNA transfection group; ^#^p < 0.05, compared with the pcDNA transfection group in the presence of lovastatin). (**d**) After 6 h treatment with 30 μM lovastatin, ChIP assay was performed. Typical trace shown are representative of four independent experiments.

Statins including lovastatin may present as histone deacetylases (HDACs) inhibitors to increase acetylation levels of cellular proteins and subsequent colorectal cancer cell death^20^. We therefore assessed whether HDACs inhibition contributes to lovastatin’s actions in FaDu cells. As show in Fig. 4a, expression of a class I HDAC, HDAC3 or a class II HDAC, HDAC4, suppressed lovastatin-induced p63 acetylation. Transfection of cells with HDAC3 or HDAC4 also reduced lovastatin-elevated p21^cip/Waf1^ levels (Fig. 4b). In addition, lovastatin-decreased survivin levels were restored in cells transfected with HDAC3 and HDAC4 (Fig. 4c). We reported previously that p63, similar to p53, might prevent the binding of Sp1 to the promoter region to reduce survivin expression in HT29 cells, a 53-mutant human colorectal cancer cell line 27. A ChIP experiment was conducted to examine whether lovastatin affects Sp1, p63 or HDAC3 binding to the putative p53/p63 and Sp1 binding sites containing promoter region (−264 to −37) of survivin. As shown in Fig. 4d, lovastatin increased p63 binding, while decreases Sp1 and HDAC3 binding to the survivin promoter region (Fig. 4d). These results suggest that HDACs inhibition contributes to lovastatin-induced p63 acetylation and subsequent cellular events in FaDu cells.

### AMPK-p38MAPK signaling in lovastatin’s actions in FaDu cells

**Figure 5:**
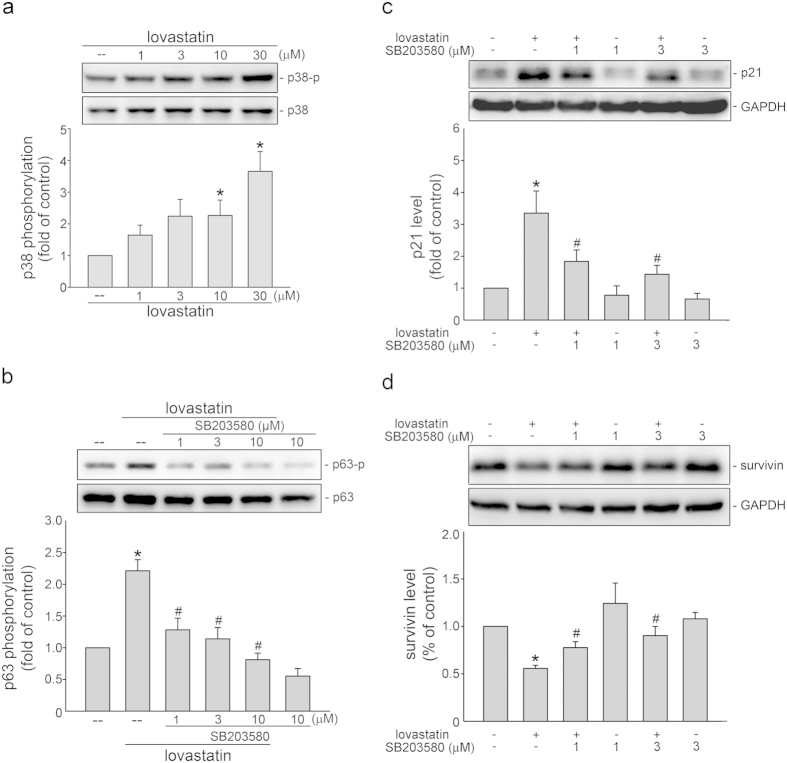
p38MAPK in lovastatin’s actions in FaDu cells. (**a**) After 6 h treatment with indicated concentrations of lovastatin, the extent of p38MAPK phosphorylation was examined by immunoblotting. Compiled results are shown at the bottom (n = 5). (*p < 0.05, compared with the control group) (**b**) Cells were treated with vehicle or SB203580 (1–10 μM) for 30 min. cells were then treated with 30 μM lovastatin for another 6 h. The extent of p63 phosphorylation was assessed by immunoblotting. Compiled results are shown at the bottom (n = 5). Cells were treated with vehicle or SB203580 (3 μM) for 30 min. Cells were then treated with 30 μM lovastatin for another 24 h. The extent of p21^cip/Waf1^ (**c**) and survivin (**d**) levels were then determined by immunoblotting. Compiled results are shown at the bottom (n = 5). *p < 0.05, compared with the control group; ^#^p < 0.05, compared with the lovastatin-treated group.

**Figure 6:**
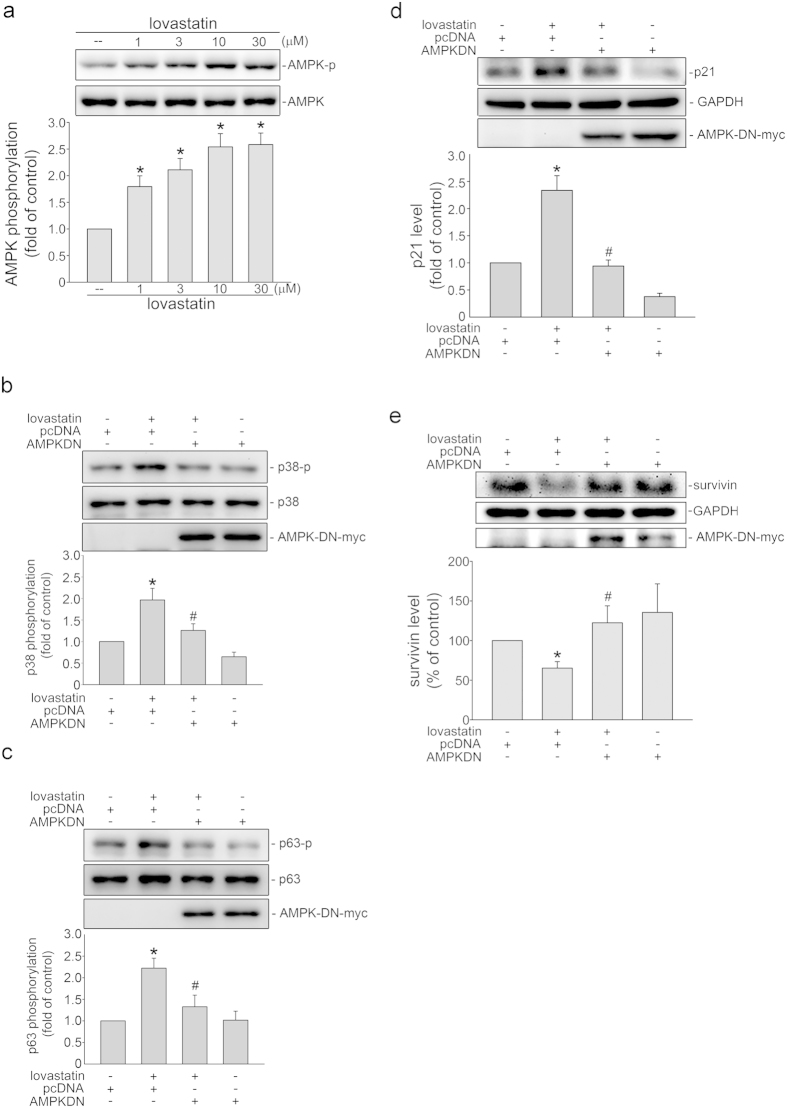
AMPK in lovastatin’s actions in FaDu cells. (**a**) After 6 h treatment with indicated concentrations of lovastatin, the extent of AMPK phosphorylation was assessed by immunoblotting. Compiled results are shown at the bottom (n = 7). Cells were treated with vehicle or 30 μM lovastatin for another 6 h after transfection. Immunoblotting was employed to determine the extent of p38MAPK (**b**) and p63 (**c**) phosphorylation. Compiled results are shown at the bottom (n = 4). Cells were treated with 30 μM lovastatin for another 24 h after transfection. Immunoblotting was employed to determine the extent of p21^cip/Waf1^ (**d**) and survivin (**e**) levels. Compiled results are shown at the bottom (n = 4). *p < 0.05, compared with the control group; ^#^p < 0.05, compared with the lovastatin-treated group.

There is increasing evidence that many apoptotic signaling cascades involve AMP-activated protein kinase (AMPK) and p38MAPK^27^,^47^. We next explored whether p38MAPK and AMPK signaling cascades contribute to lovastatin-induced FaDu cell death. As shown in Fig. 5a, lovastatin induced p38MAPK phosphorylation in FaDu cells. SB203580, a pharmacological p38MAPK inhibitor was used to determine whether lovastatin-induced p63 phosphorylation is attributable to p38MAPK activation. As shown in Fig. 5b, p38MAPK signaling blockade by SB203580 significantly reduced lovastatin-induced p63 phosphorylation. In addition, SB203580 also reduced lovastatin-elevated p21^cip/Waf1^ levels (Fig. 5c) and restored lovastatin-decreased survivin levels (Fig. 5d) in FaDu cells. Moreover, lovastatin concentration-dependently increased AMPK phosphorylation (Fig. 6a). AMPK dominant negative mutant (AMPK-DN) was employed to establish the connection between AMPK and p38MAPK signaling cascades. As shown in Fig. 6b, AMPK-DN significantly suppressed lovastatin-induced p38MAPK phosphorylation. Lovastatin-induced p63 phosphorylation was also suppressed in the presence of AMPK-DN (Fig. 6c). Moreover, lovastatin’s effecs in increasing p21^cip/Waf1^ (Fig. 6d) and decreasing survivin levels (Fig. 6e) were significantly reduced in cells transfected with AMPK-DN. These results support the fundamental role of the AMPK-p38MAPK-p63 signaling in lovastatin-induced FaDu cell death.

### NF-κB and STAT3 contribute to survivin repression in lovastatin-stimulated FaDu cells

**Figure 7:**
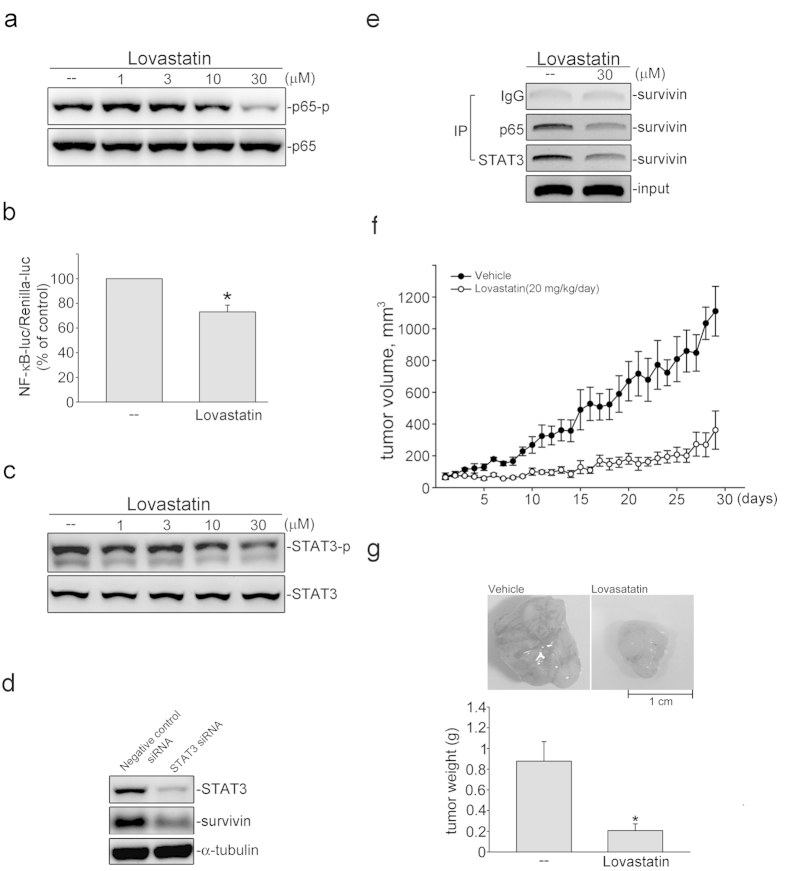
Lovastatin suppressed *in vivo* tumor growth in nude mice. (**a**) After 6 h treatment with lovastatin, p65 phosphorylation status was assessed by immunoblotting. Typical trace shown are representative of four independent experiments. (**b**) Cells were treated with lovastatin at 30 μM for another 24 h after transfection. Reporter assay was performed. Compiled results reprent the mean ± SEM of four independent experiments. (*p < 0.05, compared with the control group) (**c**) After 6 h treatment with indicated concentrations of lovastatin, STAT3 phosphorylation status was assessed by immunoblotting. Typical trace shown are representative of three independent experiments. (**d**) After transfection, the survivin, STAT3 and α-tubulin levels were determined by immunoblotting. Typical pattern shown are representative of three independent experiments. (**e**) After 6 h treatment with 30 μM lovastatin, ChIP assay was performed. Typical pattern shown are representative of three independent experiments. (**f**) Nude_nu/nu_ mice were administered intraperitoneally with vehicle or lovastatin (20 mg/kg) once daily for 29 days. Tumor volumes were also calculated daily. Results represents the mean ± S.E.M. (n = 5). (**g**) FaDu xenografts were removed and weighted at the end of the experiment. Compiled results are shown at the bottom (n = 5). *p < 0.05 as compared with the vehicle-treated control group.

In addition to p53, p63 and Sp1, the survivin promoter region (−300 to −41) also contains putative NF-κB and STAT3 binding sites. Several studies showed that transcription factors NF-κB and STAT3 play important roles in inducing survivin expression^48^. We thus determined whether NF-κB subunit p65 and STAT3 phosphorylation status, which represent NF-κB and STAT3 activation, was altered in FaDu cells after exposure to lovastatin. As shown in Fig. 7a, lovastatin reduced p65 phosphorylation in FaDu cells. Results from reporter assays showed that lovastatin reduced NF-κB-luciferase activities (Fig. 7b). Similarly, lovastatin also suppressed STAT3 phosphorylation (Fig. 7c). Transfection of cells with STAT3 siRNA significantly reduced the basal level of survivin in FaDu cells (Fig. 7d). We next determined whether lovastatin alters the recruitment of p65 or STAT3 to the *survivin* promoter region (−300 to −41). As shown in Fig. 7e 6 h exposure to lovastatin reduced p65 and STAT3 binding to the survivin promoter region. It is likely that STAT3 and NF-κB may also account for survivin repression by lovastatin in FaDu cells.

### Lovastatin suppressed tumor growth *in vivo*

We next explored the *in vivo* effects of lovastatin using a xenograft murine model. After the average tumor size of tumors reached approximately 100 mm^3^, mice were daily administrated by intraperitoneal injections (I.P.) with vehicle or lovastatin (20 mg/kg/day) for 29 days. At the end of the experiment, mice were sarcificed to collect tumor samples. As show in Fig. 7f, lovastatin reduced tumor growth comparing to the vehicle-reated control group. Mice treated with lovastatin had a smaller tumor weight (Fig. 7g). In addition, mouse body weight was not altered after lovastatin treatment (data not shown). Together, these findings suggested that lovastatin is capable of suppressing Fadu xenografts growth *in vivo*.

## Discussion

Growing evidence supports the therapeutic benefits of statins as anti-cancer agents in addition to its anti-inflammatory and anti-proliferative activities in non-cancerous tissues^1^,^49^. However, the precise anit-tumor mechanisms of statins remains incompletely understood. Keto *et al*.^50^ demonstrated that statins’ anti-tumor actions in certain tumors involved HMG-CoA reductase-mevalonate pathway. Geranyl pyrophosphate (GGPP) and farnesyl pyrophosphate (FPP) play critical roles in this pathway and are essential for activating Ras/Rho small G protein family and carcinogenesis^51^. It appears that suppression of Ras/Rho signaling by mevalonate pathway blockade contributes to statins’ anti-tumor effects. However, statins’ anti-tumor actions may also attribute to its non-lipid effects^20^,^52^. It is likely that statins’ anti-tumor effects accrue from a variety of different mechanisms. Although there have been many studies reported that statins exhibit anti-tumor properties in numerous different human cancer cell lines, few studies have been undertaken to explore the underlying mechanisms by which statins induce HNSCC cell death. We show in the present study that lovastatin causes FaDu human pharyngeal squamous carcinoma cell apoptosis via AMPK-p38MAPK-p63-survivin signaling cascade. HDACs inhibition may also be involved in lovastatin’s actions in FaDu cells.

Activated AMPK regulates cell survial and growth by activating the downstream signaling events, including p38MAPK activation^27^ and Akt-mammalian target of rapamycin (mTOR) pathway down-regulation^53^. Whether p38MAPK contributes to lovastatin-induced FaDu cell death has not been previously reported. We show in this study that p38MAPK activation was causally related to lovastatin’s actions. We also demonstrated that AMPK mediated lovastatin’s effects on p38MAPK activation and survivin modulation in FaDu cells. However, whether lovastatin-activated AMPK leads to autophagy in FaDu cells, as suggested in another study^54^, remain to be established. The precise mechanism by which lovastatin induces AMPK phosphorylation in FaDu cells remain unresolved. Increased intracellular AMP/ATP ratio might play a causal role in AMPK activation and cell death^16^. Activation of tumor suppressor LKB1, a putative AMPK kinase, also contributed to lovastatin-induced AMPK activation in SCC cells^16^. Moreover, Kou *et al*.^55^ demonstrated that simvastatin-induced LKB1 phosphorylation is a consequence of mevalonate-Rac1 cascade activation. However, knockdown of Rac1 did not affect simvastatin-induced AMPK phosphorylation in endothelial cells^55^. Together these observations suggest that increased AMP/ATP ratio may contribute to lovastatin-activated AMPK-p38MAPK apoptotic signaling cascade in FaDu cells. Whether lovastatin affects AMP/ATP ratio in FaDu cells exposed remains to be investigated. It is also worthy to clarify whether mevalonate pathway or LKB1-related mechanisms contributes to lovastatin-induced AMPK activation and subsequent cellular events in FaDu cells.

Lovastatin was previously shown to induce SCC cell death^16^. Kaneco *et al*.^56^ further reported that survivin down-regulation contributes to lovastatin-induced colorectal cancer cell cell death. Similarly, we showed that survivin repression by lovastatin led to FaDu cell apoptosis. We further demonstrated the effectiveness of lovastatin in suppressing tumor progression in an *in vivo* xenograft murine model. The underlying mechanisms by which lovastatin induces survivin down-regulation and apoptosis in FaDu cells remains incompletely understood. We noted that knock-down p63 using p63 siRNA restored lovastatin’s effects of decreasing survivin. It appears that p63 is causally related to lovastatin-induced survivin repression. Activation of p63 is regulated by its modifications such as phosphorylation, acetylation and ubiquitination^42^,^44^. In this study, we showed that AMPK-p38MAPK signaling blockade reduced lovastatin-induced p63 phosphoryaltion. These results support the contention that lovastatin activates the AMPK-p38MAPK-p63 pathway, leading to survivin down-regulation and subsequent cell death in FaDu cells.

Elevated levels of HDAC family members in tumor cells are correlated with poor prognosis in cancer patients^57^,^58^. Lin *et al*.^20^ reported that statins may induce cancer cell death via HDACs inhibition. In agreement with this, we showed in this study that expression of HDAC3 or HDAC4 significantly reduced lovastatin’s actions on p21^cip/Waf^ and survivin levels. Moreover, HDAC3 and HDAC4 also attenuated lovastatin-increased p63 acetylation. It is likely that p63 modified by phosphorylation and acetylation may contribute to lovastatin-induced p63 activation in FaDu cells. Whether lovastatin-induced p63 acetylation involves other HDAC isoforms remains to be investigated.

Similar to our previous report that p38MAPK-p53-survivin signaling mediated simvastatin-induced HCT116 colorectal cancer cell death^6^, we demonstrated that AMPK-p38MAPK cascade also plays a pivotal role in lovastatin-induced FaDu hypopharyngeal carcinoma cell death. In contrast to p53, we showed that p63, contributes to survivin repression and cell death in p53 mutant FaDu cells. These findings suggest that p38MAPK and p53 family members may play pivotal roles in statins-induced cancer cell death. Moreover, statins was reported to inhibit renal cancer cell proliferation and metastasis through inactivating STAT3 signaling^59^. It also suppresses NF-κB-dependent anti-apoptotic gene expression to promote cell apoptosis^60^. Consistent with this, we noted in this study that lovastatin suppressed STAT3 and NF-κB activation and reduced their binding to the survivin promoter region in FaDu cells. Whether lovastatin affects the interactions between p63 and these transcription factors to localize within the survivin promoter needs further investigations.

In conclusion, we show that lovastatin exhibits anti-tumor properties, at least in part, via AMPK-p38MAPK-p63-survivin signaling cascade in FaDu cancer cells. Moreover, lovastatin also suppressed the phosphorylations of ERK1/2 and Akt, the survival signaling molecules that causally related to NF-κB and STAT3 activation, in FaDu cells (unpublished data). The exact mechanisms of these activities remain to be fully investigated, but together these observations support the therapeutic potential of lovastatin in future oncologic therapy in HNSCC patients.

## Materials and Methods

### Reagents

Lovastatin was purchased from Calbiochem (San Diego, CA, USA). TrypLE™, fetal bovine serum (FBS), streptomycin, penicillin, and MEM medium were obtained from Invitrogen (Carlsbad, CA, USA). Antibodies against AMPK, AMPK phosphorylated at Thr172, acetylated-lysine, p63, p63 phosphorylated at Ser160/Ser162, STAT3 phosphorylated at Tyr705, caspase 3, PARP and survivin were ontained from Cell Signaling (Beverly, MA, USA). Antibodies against p40 (p63 delta) and α-tubulin were obtained from Novus Biologicals (Littleton, CO, USA). Antibodies against p65, STAT3, HDAC3, Sp1, p21^cip/Waf1^, and normal IgG and were from Santa Cruz Biotechnology (Santa Cruz, CA, USA). Antibodies against Myc-tag, DDDDK (Flag), p65 phosphorylated at Ser536, and cyclin D1, as well as secondary antibodies were purchased from GeneTex Inc (Irvine, CA, USA). Dr. Eric Verdin (Department of Medicine, University of California, San Francisco, USA) kindky provided flag-tagged HDAC3 (Addgene plasmid 13819) and HDAC4 (Addgene plasmid 13821) constructs as described previously^61^. Dr. Morris Birnbaum (HHMI, PA, USA) kindly provided AMPK dominant negative mutant (AMPK-DN). ECL detection kit and transfection reagent, Turbofect^TM^ were from Millipore (Billerica, MA, USA). Dual-Glo luciferase assay system and reporter constructs including NF-κB-Luc and renilla-luc were purchased from Promega (Madison, WI, USA). All materials for immunoblotting were purchased from GE Healthcare (Little Chalfont, UK). All other chemicals were from Sigma-Aldrich (St Louis, MO, USA).

### Cell culture

Human hypopharyngeal carcinoma FaDu cell line was obtained from the Bioresource Collection and Research Center (BBRC, Hsinchu, Taiwan). FaDu cells were cultured in sodium pyruvate (1 mM), streptomycin (100 μg/ml), penicillin G (100 U/ml) and 10% FBS containing MEM medium in a humidified 37 °C incubator.

### Immunoblotting

Immunoblotting was conducted as described previously^6^. Cells were lysed using tris (10 mM, pH 7.0), triton X-100 (1%), pepstatin A (0.05 mM), leupeptin (0.2 mM), NaCl (140 mM), MgCl_2_ (1 mM) and PMSF (2 mM) containing lysis buffer. Equal amount of each sample was subjected to SDS-PAGE. The protein was then transferred to the nitrocellulose membrane. After blocking for 1 h, target protein was detected by incubating in the specific primary antibody solution for 2 h and in the secondary antibody solution for another 1 h. Target proteins were visualized and quantified using ECL detection kit and densitometer with a scientific imaging system (Biospectrum AC System, UVP, Upland, CA, USA) .

### MTT assay

To determine cell viability, the colorimetric MTT assay was employed as described previously^6^.

### Flow cytometric analysis

Flow cytometric analysis with propidium iodide (PI) single staining was performed using FACS Calibur and Cellquest program (BD Biosciences, San Jose, CA, USA) as described previously^6^. The percentage of cell cycle distribution was analyzed using ModFit programs (BD Biosciences, San Jose, CA, USA). The annexin V-FITC and PI double labeling was also employed to detect apoptotic cells. After treatment, cells were incubated for 15 min in the staining buffer (2 μg/ml annexin V-FITC, 40 μg/ml PI). The FACSCalibur and Cellquest program was employed to analyze the samples. The FCS Express program (BD Biosciences, San Jose, CA) was used to determine the percentage of stained cells in three quadrants: the lower left (annexin V^−^PI^−^) quadrant, which reprents the viable cells; the lower right (annexin V^+^PI^−^) quadrant, which represents the early apoptotic cells; the upper right (annexin V^+^PI^+^) quadrant, which reprents advanced apoptotic and necrotic cells.

### RT-PCR (reverse-transcription polymerase chain reaction) analysis

TRIzol reagent (Thermo Fisher Scientific, Waltham, MA, USA) and GoScript™ reverse transcription system (Promega , Madison, WI, USA) were used to isolate total RNA and perform reverse transcription. Primers used to generate 187 bp survivin fragment and 420 bp GAPDH fragment are: survivin sense, 5′-gcc ttt cct taa agg cca tc-3′; survivin anti-sense, 5′-aac cct tcc cag act cca ct-3′; GAPDH sense, 5′-gtc agt ggt gg acct gac ct-3′; GAPDH anti-sense, 5′-agg ggt cta cat ggc aac tg-3′. The PCR reaction with 25 cycles (30 s denature at 94 °C, 30 s annealing at 56 °C, 45 s extension at 72 °C) of amplification was performed. Amplication products were subjected to agarose gel electrophoresis and detected using ethdium bromide staining and ultraviolet illumination.

### Transfection in FaDu cells

FaDu cells (7 × 10^4^ cells per well) were transfected with flag-tagged HDAC3 or HDAC4, AMPK-DN or pcDNA for immunoblotting or transfected with *survivin* siRNA, STAT3 siRNA or negative control siRNA for flow-cytometric analysis using Turbofect^TM^ transfection reagent (Millipore, Billerica, MA, USA).

### Reporter assay

FaDu cells were transfected for 24 h with NF-κB-luc and renilla-luc reporter constructs using Turbofect^TM^ transfection reagent (Millipore, Billerica, MA, USA). After treatment with vehicle or lovastatin (30 M) for another 24 h, cells were harvested, and the luciferase activity was examined using a Dual-Glo luciferase assay system kit (Promega, Madison, WI, USA). The renilla luciferase activity represents as the basis for normalization.

### Suppression of survivin or STAT3 expression

Target gene suppression was performed as previously described^6^. For *survivin* and *STAT3* suppression, pre-designed siRNAs targeting the human *survivin (BIRC5)* and *STAT3* were from Sigma-Aldrich (St Louis, MO, USA). The siRNA oligonucleotides were as follows: *survivin* siRNA, 5′-ccucuacuguuuaacaaca-3′ and *STAT3* siRNA, 5′-ggauaacgucauuagcaga-3′. The negative control siRNA was also from Sigma-Aldrich (St Louis, MO, USA).

### Immunoprecipitation

Cells were lysed in 0.5 ml lysis buffer (1 mM PMSF, 1% Triton X-100, 10 μg/ml leupeptin, 10 μg/ml aprotinin, 100 μM sodium orthovanadate, 20 mM Tris-HCl, pH 7.5, 1 mM MgCl_2_ and 125 mM NaCl). After centrifugation for 30 min at 4 °C, the supernatant was removed and incubated with antibodies against IgG or p63 with gentle rotation at 4 °C overnight. To collect the immune complexes, 15 μl protein A-Magnetic Beads (Millipore) was added at 4 °C for another 2 h. After washed with lysis buffer for three times, the immunoprecipitated complexes were subjected to immunoblotting for assessing acetylation status of p63.

### ChIP (Chromatin immunoprecipitation) analysis

The ChIP analysis was conducted as previously described^6^. The 262-bp and 228-bp *survivin* promoter fragments between −302 and −41 or −264 and −37 were amplified using the following primers: sense-1, 5′-GATTACAGGCGTGAGCCACT-3′ and antisense-1, 5′-ATCTGGCGGTTAATGGCGCG-3′; sense-2, 5′-TTCTTTGAAAGCAGTCGAGG-3′; antisense-2, 5′-TCAAATCTGGCGGTTAATGG-3′. The PCR reaction with 30 cycles (30 s denature at 94 °C, 30 s annealing at 56 °C, 45 s extension at 72 °C) of amplification was performed. The PCR products were subjected to agarose gel electrophoresis and detected using ethdium bromide staining and ultraviolet illumination.

### *In vivo* xenograft mouse model

4-week old nude_nu/nu_ mice (BioLasco, Taipei, Taiwan) were used to perform xenograft model. PBS in a volume of 300 μl containing FaDu cells (5 × 10^6^ cells) were injected subcutaneously into the flank of each mouse. Mice were treated with vehicle or lovastatin (20 mg/kg/day) after the tumor size reached approximately 100 mm^3^. Lovastatin was intraperitoneally administered once daily for 25 days. A digital caliper was used to measure the tumor size every day. The formula *V* (mm^3^) = [*ab*^2^]× 0.52 (*a*: the length of the tumor; *b:* the width of the tumor) was used to calculate tumor volume. At the end of the treatment, mice were sacrificed to remove xenografts. These procedures were approved (Permit Number: LAC-2014-0230) by the Taipei Medical University Laboratory Animal Care and Use Committee. The present study was performed in strict accordance with the recommendations in the Guide for the Care and Use of Laboratory Animals of the National Institutes of Health and in accordance with the approved guidelines. All surgery was conducted under sodium pentobarbital anesthesia to minimize suffering.

### Statistical analysis

Compiled results represent as the mean ± S.E.M. of at least three independent experiments. To determine the statistical significance of the difference between means, one-way analysis of variance (ANOVA) and the Newman-Keuls test were used, when appropriate. It is considered statistically significant when a *p* value of <0.05.

## Additional Information

**How to cite this article**: Yen, C.-S. *et al*. Lovastatin causes FaDu hypopharyngeal carcinoma cell death via AMPK-p63-survivin signaling cascade. *Sci. Rep*. **6**, 25082; doi: 10.1038/srep25082 (2016).

## Supplementary Material

Supplementary Information
