# Supplementary material for: Lovastatin causes FaDu hypopharyngeal carcinoma cell death via AMPK-p63-survivin signaling cascade
Source: Sci Rep. 2016 Apr 28;6:25082. doi: 10.1038/srep25082 (PMC4848532; doi:10.1038/srep25082)
Supplement: Supplementary Information [file srep25082-s1.pdf]

Lovastatin causes FaDu hypopharyngeal carcinoma cell death via AMPK-p63-survivin signaling cascade

Chia-Sheng Yen[1#], Jung-Chien Chen[2#], Yi-Fang Chang[3], Ya-Fen Hsu[4], Pei-Ting Chiu[5], Ching Shiue[4], Yu-Fan Chuang[5], George, Ou[6], Ming-Jen Hsu[5,7*]

[1]Department of General Surgery, Chi-Mei Medical Center, Tainan, Taiwan; [2]Division of General Surgery, Department of Surgery, Min-Sheng General Hospital, Taoyuan, Taiwan; [3]Division of Hematology and Oncology, Department of Internal Medicine, MacKay Memorial Hospital, Taipei, Taiwan; [4]Division of General Surgery, Department of Surgery, Landseed Hospital, Taoyuan, Taiwan; [5]Graduate Institute of Medical Sciences, College of Medicine, Taipei Medical University, Taipei, Taiwan; [6]Department of Medicine, University of British Columbia, Vancouver, British Columbia, Canada; [7]Department of Pharmacology, School of Medicine, College of Medicine, Taipei Medical University, Taipei, Taiwan

[#]These authors contributed equally to this study.

[*]Correspondence should be addressed to:

Dr. Ming-Jen Hsu, Department of Pharmacology, School of Medicine, Taipei Medical University, No.250, Wu-hsing St., Taipei 11031, Taiwan; Tel: +886-2-27361661 ext. 3198; E-mail: aspirin@tmu.edu.tw

Supplementary Figure 1

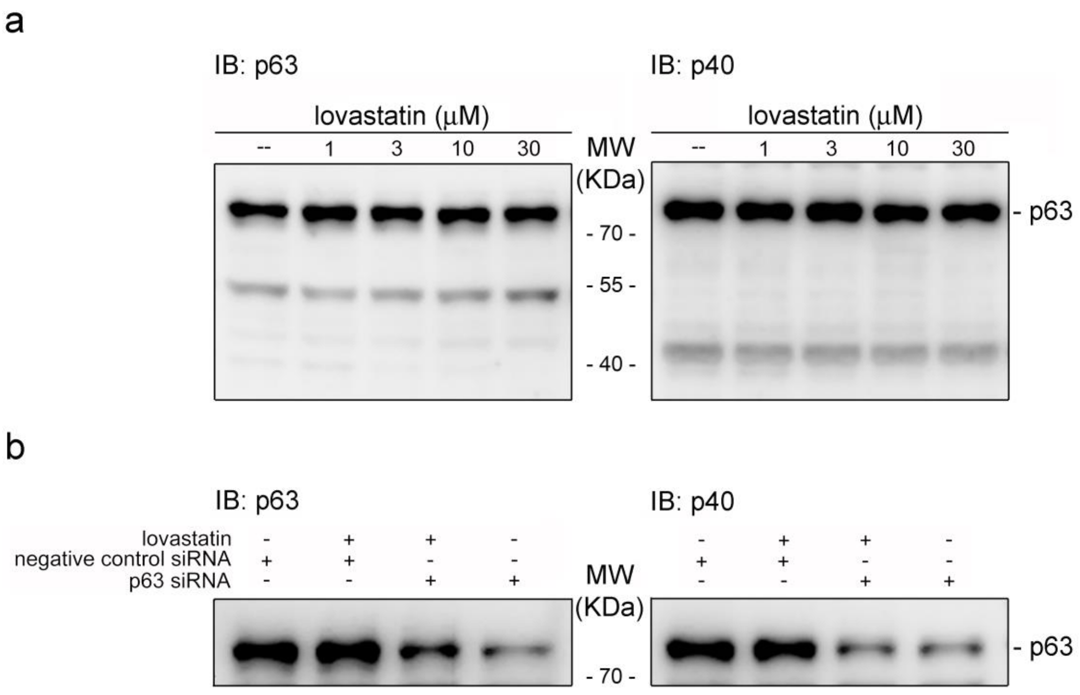

Supplementary Figure 1. Anti-p63 antibody recognizes the same protein (p63) as anti-p40 antibody does in FaDu cells. (A) Cells were treated with lovastatin at indicated concentrations for 6 h. Cell lysates were then prepared and subjected to immunoblotting with anti-p60 antibody or anti-p40 antibody. Results shown are representative of three independent experiments. (B) Cells were transfected with negative control siRNA or p63 siRNA for 48 h. Protein level of p63 was then determined by immunoblotting using anti-p60 antibody or anti-p40 antibody. Results shown are representative of three independent experiments. IB: immunoblotting; MW: molecular weight
